# Frequency of Oral Squamous Cell Carcinoma in Two Public Hospitals in Peru: A Ten-Year Retrospective Study

**DOI:** 10.3290/j.ohpd.c_2406

**Published:** 2025-12-12

**Authors:** Mariafernanda Villar-Rivera, Patricia Esther Asian-Suarez, Javier Bernardo Cruz-Colca, Julissa Amparo Dulanto-Vargas

**Affiliations:** a Mariafernanda Villar-Rivera Graduate Student, School of Dentistry, Universidad Científica del Sur, Lima, Peru. Idea, study design, data curation, formal analysis, original draft preparation.; b Patricia Esther Asian-Suarez Assistant Professor, School of Dentistry, Universidad Científica del Sur, Lima, Peru. Idea, study design, supervision, reviewed the manuscript.; c Javier Bernardo Cruz-Colca Assistant Professor and Specialist in Head, Neck and Maxillofacial Surgery, Hospital Nacional Guillermo Almenara Irigoyen, Head and Neck Surgery Service, School of Dentistry, Universidad Científica del Sur, Lima, Peru. Idea, study design, supervision, reviewed the manuscript.; d Julissa Amparo Dulanto-Vargas Research Associate, Research Group in Dental Sciences, School of Dentistry, Universidad Científica del Sur, Lima, Peru. Idea, study design, formal analysis, reviewed and edited the manuscript.

**Keywords:** epidemiology, oral medicine, squamous cell carcinoma.

## Abstract

**Purpose:**

To determine the frequency and factors associated with oral squamous cell carcinoma (OSCC) in the Peruvian population.

**Materials and Methods:**

This retrospective study (2013 to 2022) included data from clinical records of 416 patients from the Head-and-Neck and Oncology Services in two public hospitals in Lima. The primary variable included the presence, location, and degree of differentiation of OSCC with a confirmatory diagnosis from anatomy pathology. Secondary variables included demographic and health data. Comparisons were analyzed using prevalence ratios (PR) with 95% confidence intervals (CI) and statistical significance set at p < 0.05.

**Results:**

OSCC was identified in 169 cases (40.6%; 95% CI: 35.9‒45.4). Localization was frequently on the tongue (lateral border 34.5%, mobile 9.5% and base 7.1%), with a well- or moderately differentiated grade (97.4%). The presence of OSCC was similar in both sexes (1.2:1 ratio), was more prevalent in individuals aged 51 to 80 years (66.9%), retired (40.1%), born on the coast (66.7%) and diagnosed in 2018 and 2019 (30.8%). The PRs of OSCC were statistically significantly higher in men (palate: PR 2.77), patients >50 years (presence PR 1.54, lip: 5.28, moderately and well-differentiated: PR 1.11), retired persons (lip: PR 5.52), and those born in the highlands (lip: PR 2.4) (p ≤ 0.04).

**Conclusion:**

OSCC was frequent in cases of suspected oral cancer, and more frequently affected the tongue, was well- or moderately differentiated, and associated with the demographic factors of sex, age, occupation, and region of birth.

Oral cancer (OC) ranks 16th among all cancers and is the third most common neoplasm of the head and neck, with more than 389 thousand new cases worldwide, according to the Global Cancer Observatory (GLOBOCAN) in 2022. It is estimated to cause 1.9 of all cancer deaths worldwide with an average of 188 thousand deaths.^26^ This neoplasm has shown an increasing global trend and has a significant impact on regions such as Latin America with limited access to healthcare systems.^49^ According to the latest report from the Centers for Disease Control and Prevention (CDC) in Peru, the number of people affected by OC increased from 143 cases in 2021 to 220 cases in 2024, with an average of 25.7 new cases per year among patients treated in hospitals affiliated with the Peruvian Ministry of Health.^15^

In 90% of individuals, oral cavity cancer presents as oral squamous cell carcinoma (OSCC).^22^ This variant originates from the epithelium in a warty, in-situ, pigmented and hybrid form.^8^ Signs and symptoms include exophytic lesions or potentially malignant ulcerations, although these lesions do not usually appear in early stages. Diagnosis of OSCC is usually achieved by clinical examination and tissue biopsy.^17,35,38^ The identification and location of OSCC has not recently been reported in public hospitals in Peru. However, reports on OC from 2021 to 2024 indicate that it was mainly found in the oral cavity (48% to 49.7%), tongue (42.7% to 45.4%), and lips (4.9% to 9.1%), and primarily affected women (48.3% to 58.2%) and residents of the capital city of Lima (12.6% to 21.6%).^15^

OSCC affects different tissues depending on the degree of keratinization. Differentiation depends on the type of carcinogen exposure, making surgical treatment challenging.^22,39^ Tobacco, alcohol and human papillomavirus (HPV) are reported to be common risk factors for OSCC.^2,22,27,29^ Premalignant oral lesions, such as leukoplakia, erythroplakia, and submucous fibrosis, can indicate early signs of OSCC.^27^ On the other hand, the stage of OSCC has prognostic value for treatment decision making, with the treatment of choice being surgical excision in early stages, followed by adjuvant radiotherapy and chemotherapy in advanced stages.^10,22,27^

Previous studies using institutional or governmental health data found from 124 to 33,619 cases of OSCC in 35 populations. OSCC was mainly located on the tongue and with a well-differentiated histology. Demographic data showed a higher prevalence in males and from the fifth decade of life onwards (supplementary Table A1).^1,3-7,9,13,14,16,18-21,23-25,30,32-34, 36,37,40,41,43,44,46,50^ A multi-center study of OSCC patients at a private university dental clinic in Lima, spanning the period from 1982 to 2022, identified 540 cases, with a higher prevalence among females (55.2%), older age groups (average age 64.8 years), and tumors located on the tongue (50%), specifically the lateral border (28.1%), with a predominantly well-differentiated grade (84.3%).^25^ Disparities in lifestyles and health determinants by region can increase the burden and prognosis of this disease.^22^

More deaths are caused by OSCC than other conditions involving the orofacial region. Peru has seven public health institutions for specialized care of patients with OC, most of whom are referred from primary care. It is necessary to understand the role of demographic and health factors that may be associated with OSCC in order to achieve early diagnosis and improve survival. Therefore, the aim of this study was to determine the frequency and factors associated with OSCC in two public hospitals in Peru. The study hypothesis was that the incidence of OSCC is increasing over time, and that its presence, location, and degree of differentiation are associated with the demographic characteristics of the individuals affected.

## MATERIALS AND METHODS

### Study Design and Ethics Approval

This retrospective study was approved by the Ethics Committee of the Universidad Científica del Sur (N°113-CIEI-CIENTÍFICA-2023), Santa Rosa Hospital (No. 138-2020-DG-HSR-MINSA) and Guillermo Almenara Irigoyen Hospital (N°346-CIEI-OIyD-GRPA-ESSALUD-2023). The Santa Rosa Hospital is affiliated with the Ministry of Health (MINSA) and the Guillermo Almenara Irigoyen Hospital is affiliated with the Health Social Security program (EsSalud). The study was conducted in accordance with the principles of the Declaration of Helsinki and the STROBE guidelines.

### Study Population

The data source consisted of 416 medical records of patients aged 20 to 90 years (189 males, 227 females) who were treated by the Head-Neck and Oncology Services of two public hospitals. The data corresponded to the period from January 2013 to December 2022 provided by the Statistics Departments of the Santa Rosa Hospital (n = 113) and the Guillermo Almenara Irigoyen Hospital (n = 303). Both hospitals are located in Lima and have the highest level of care in the country (levels III-1 and III-2, respectively), including evaluation of rare health problems by specialized medical-surgical services.

### Selection Criteria

The inclusion criteria were electronic and physical medical records with anatomy-pathology reports of head and neck tissue biopsies. Exclusion criteria were physical medical records with unintelligible data and electronic medical records that could not be opened in the system (n = 60). Data collection from the medical records was performed by a single observer (MVR) who was previously trained by two experts (PEAS and JBCC) on OSCC codes and evaluated up to 40 medical records per day.

### Primary Variable

**Fig 1a to e:**
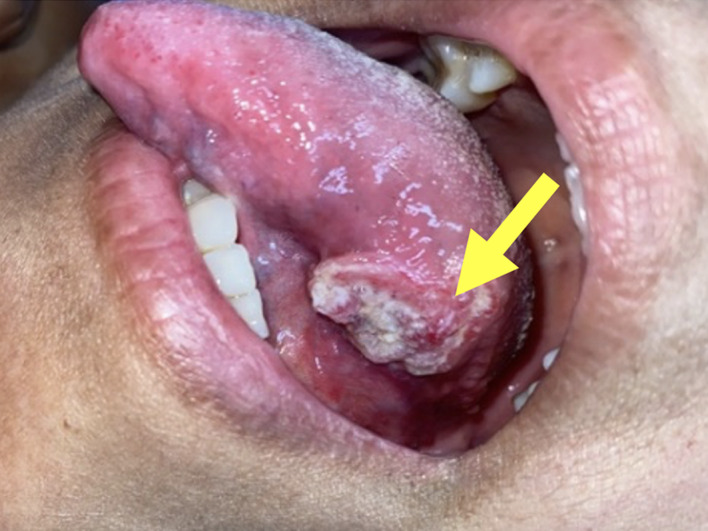

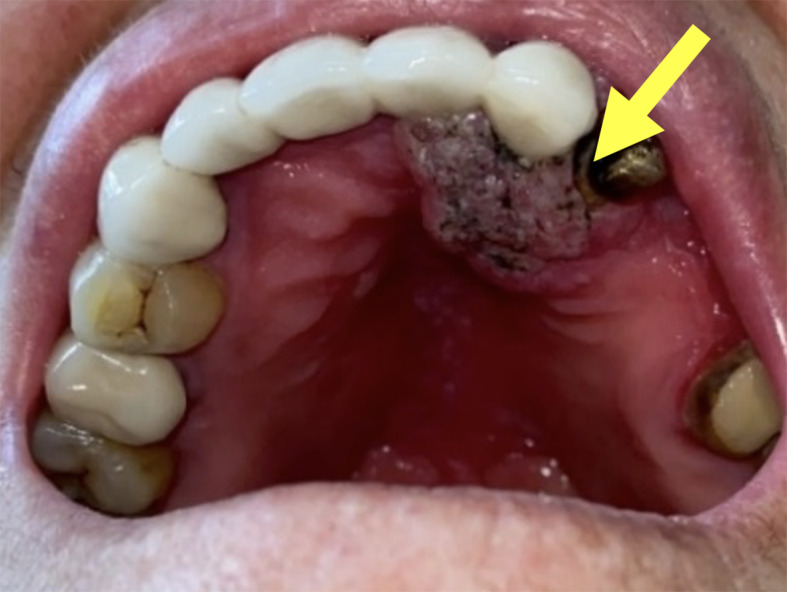

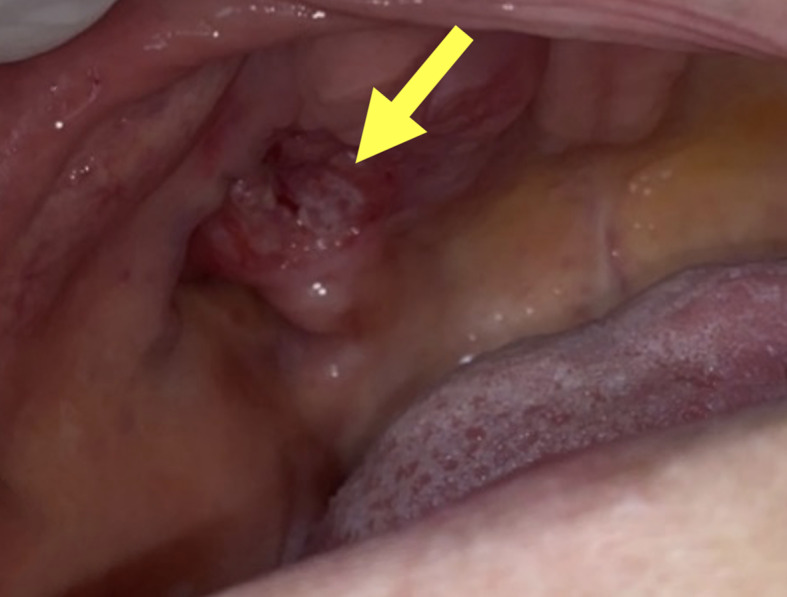

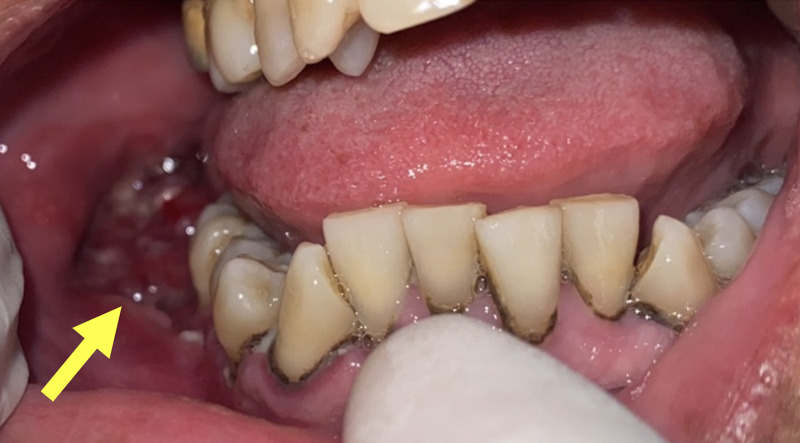

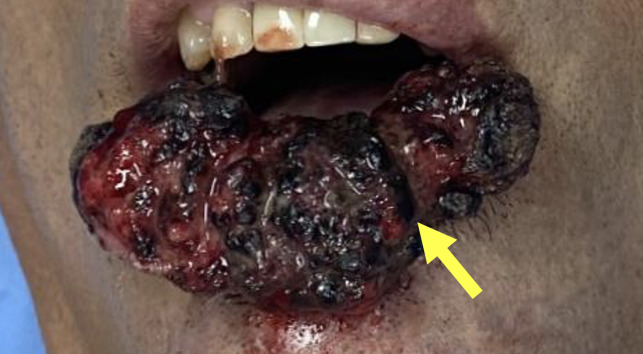
Clinical images of oral squamous cell carcinoma in: (a) Lateral border of the tongue: exophytic tumor in the posterior third of the left border of the tongue, with irregular borders, maintaining adequate mobility of the tongue. (b) Palate: exophytic tumor with irregular borders extending to the gingiva in upper incisor teeth. (c) Upper gingiva: ulcerated exophytic tumor of the right upper vestibular sulcus extending to the palate in a senile edentulous patient with a long-standing movable prosthesis. (d) Retromolar trigone: ulcerated exophytic tumor of the mucosa of the left retromolar trigone extending to the jugal mucosa in a patient with limited oral opening due to trismus, reflecting the infiltration of the tumor into the masticatory muscles. (e) Lip: large exophytic tumor extending along the lower lip with ulcerations that bleed easily on contact. Source: photographs provided by Hospital Guillermo Almenara Irigoyen.

**Fig 2a to f:**
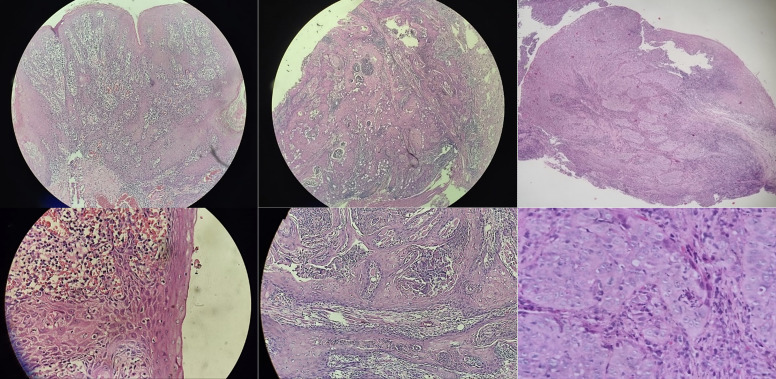
Histopathological images with hematoxylin and eosin staining to evaluate the degree of differentiation of oral squamous cell carcinoma. (a) Well differentiated: similar tumor and squamous cells. (b) Moderately differentiated: different, but recognizable, tumor and squamous cells. (c) Poorly differentiated: different tumors and squamous cells. (a1) At 10X magnification there is irregular infiltration of epithelial cells invading the connective tissue, showing deposits of keratin. (a2) At 40X magnification, infiltration of malignant epithelial cells into the connective tissue with clear nuclear pleomorphism and hyperchromatism and some areas of hemorrhagic foci are observed. (b1) At 20X magnification, infiltration of epithelial cells grouped in nests with clear pleomorphism is observed. (b2) At a higher magnification of 40Xthe clusters of neoplastic cells in nests are more clearly seen. (c1) At 10X magnification an infiltrate of malignant epithelial cells is seen throughout the connective tissue forming multiple nests. (c2) At 40X magnification high cellular differentiation of atypical epithelial cells and presence of nuclear pleomorphism and multiple mitoses are seen. Source: photographs provided by Hospital Guillermo Almenara Irigoyen.

OSCC was analyzed according to the International Classification of Diseases (ICD-10) registry of malignant neoplasms of the lip and oral cavity, which includes malignant tumors of the lip (C00), base of tongue (C01), other unspecified parts of the tongue (C02), gingiva (C03), base of the mouth (C04), palate (C05) and other unspecified parts of the tongue/mouth (C06). The registration of OSCC was limited to cases confirmed by anatomy-pathology tissue biopsy examination. Primary variables concerning OSCC were adapted from hospital clinical records, including data on frequency (presence or absence), location (movable tongue, base of tongue, floor tongue line, lateral border of tongue, retromolar trigone, palate, floor of mouth, gingiva, upper lip, lower lip and lip mucosa) (Fig 1) and grade of differentiation ‒ undifferentiated, poorly differentiated, moderately differentiated, well-differentiated and indeterminate ‒ adopted by the World Health Organization Classification based on Broder’s criteria (Fig 2).^46^

### Secondary Variables

Demographic data as well as information related to health habits and hospital care were collected from medical records, which included the following secondary variables: sex (male, female), age recorded according to seven ranges (≤30 years, 31 to 40 years, 41 to 50 years, 51 to 60 years, 61 to 70 years, 71 to 80 years, ≥81 years), occupation (bricklayer, miner, retired, housewife, student, doctor, driver, employee, self-employed, technician, teacher), birth location (coast, highlands, and jungle region), current or past smoking and alcohol consumption (no, yes), year of tumor detection (2013 to 2022). Missing data were noted as not recorded (codes 9 or 99).

### Statistical Analysis

Descriptive statistics included frequencies and percentages. Primary (location and differentiation) and secondary variables (demographic variables) were regrouped for prevalence ratios (PR) and confidence interval (CI) analysis. Data were analyzed with Jamovi v.2.6.26 statistical software (JASP, University of Amsterdam) at p < 0.05.

## RESULTS

OSCC was found in 169 cases (40.6%), with suspected OC (95% CI: 35.9%‒45.4%) being similar between females and males (1.2:1 ratio), and more prevalent in individuals aged 51 to 80 years (66.9%), retired persons (40.1%), those born on the coast (66.7%), and detected in 2018/2019 (30.8%). Most of the patients with OSCCs had no history of tobacco (66.7%) or alcohol (70.7%) use (Table 1).

**Table 1:** Distribution of the presence of oral squamous cell carcinoma according to the demographic and health characteristics of the study sample

Variables	Items	Total	No	Yes
n	%	n	%	n	%
Presence		416	100.0%	247	59.4%	169	40.6%
Sex (n = 169)	Male	189	45.4%	112	45.3%	77	45.6%
Female	227	54.6%	135	54.7%	92	54.4%
Age (n = 169)	20–30 years	20	4.8%	14	5.7%	6	3.6%
31–40 years	21	5.0%	17	6.9%	4	2.4%
41–50 years	59	14.2%	40	16.3%	19	11.2%
51–60 years	83	20.0%	46	18.8%	37	21.9%
61–70 years	86	20.7%	51	20.8%	35	20.7%
71–80 years	83	20.0%	42	17.1%	41	24.3%
81-90 years	62	14.9%	35	14.3%	27	16.0%
Occupation (n = 137)	Bricklayer	10	2.4%	6	3.4%	4	2.9%
Miner	2	0.5%	1	0.6%	1	0.7%
Retired	122	29.3%	67	37.9%	55	40.1%
Housewife	80	19.2%	48	27.1%	32	23.4%
Student	13	3.1%	9	5.1%	4	2.9%
Doctor	3	0.7%	1	0.6%	2	1.5%
Driver	6	1.4%	4	2.3%	2	1.5%
Employee	22	5.3%	13	7.3%	9	6.6%
Self-employed	34	8.2%	17	9.6%	17	12.4%
Technician	3	0.7%	3	1.7%	0	0.0%
Teacher	19	4.6%	8	4.5%	11	8.0%
Birthplace (n = 147)	Coast	253	60.8%	155	71.8%	98	66.7%
Highlands	100	24.0%	57	26.4%	43	29.3%
Jungle	10	2.4%	4	1.9%	6	4.1%
Tobacco (n = 60)	No	99	23.8%	59	74.7%	40	66.7%
Yes	40	9.6%	20	25.3%	20	33.3%
Alcohol (n = 58)	No	96	23.1%	55	72.4%	41	70.7%
Yes	38	9.1%	21	27.6%	17	29.3%
Hospital (n = 169)	Level III-1	113	27.2%	75	30.4%	38	22.5%
Level III-2	303	72.8%	172	69.6%	131	77.5%
Year of detection (n = 169)	2013	17	4.1%	9	3.6%	8	4.7%
2014	17	4.1%	12	4.9%	5	3.0%
2015	39	9.4%	25	10.1%	14	8.3%
2016	42	10.1%	27	10.9%	15	8.9%
2017	39	9.4%	23	9.3%	16	9.5%
2018	62	14.9%	38	15.4%	24	14.2%
2019	67	16.1%	39	15.8%	28	16.6%
2020	39	9.4%	20	8.1%	19	11.2%
2021	43	10.3%	24	9.7%	19	11.2%
2022	51	12.3%	30	12.1%	21	12.4%


The localization of OSCC was mainly on the tongue (50.9%) (lateral border 34.5%, mobile 9.5% and base 7.1%), followed by the palate (13.7%), lower lip (12.5%) and oral mucosa (10.1%). Cases of OSCC of the lateral border of the tongue were more frequent in females (63.8%) compared to males (36.2%). Cases of OSCC of the tongue (base and lateral border), palate, floor of mouth, gingiva and lower lip were more frequent in level III-2 hospital care (79.3 to 100%) compared to level III-1 (< 20.7%) (Table 2).

**Table 2:** Distribution of the location of oral squamous cell carcinoma according to the demographic and health characteristics of the study sample

Variables	Items	Dorsum of the tongue	Tongue base	Lateral border of the tongue	Retromolar trigone	Palate	Floor of the mouth	Gum	Upper lip	Lower lip	Oral mucosa
n	%	n	%	n	%	n	%	n	%	n	%	n	%	n	%	n	%	n	%
Total (n = 168)		16	9.5%	12	7.1%	58	34.5%	2	1.2%	23	13.7%	6	3.6%	11	6.5%	2	1.2%	21	12.5%	17	10.1%
Sex (n =168)	Male	5	31.3%	8	66.7%	21	36.2%	0	0.0%	16	69.6%	2	33.3%	8	72.7%	0	0.0%	10	47.6%	6	35.3%
Female	11	68.8%	4	33.3%	37	63.8%	2	100.0%	7	30.4%	4	66.7%	3	27.3%	2	100.0%	11	52.4%	11	64.7%
Age in years (n = 168)	20–30 years	0	0.0%	0	0.0%	2	3.4%	0	0.0%	2	8.7%	1	16.7%	0	0.0%	0	0.0%	0	0.0%	1	5.9%
31–40 years	1	6.3%	0	0.0%	2	3.4%	0	0.0%	0	0.0%	0	0.0%	1	9.1%	0	0.0%	0	0.0%	0	0.0%
41–50 years	1	6.3%	1	8.3%	11	19.0%	0	0.0%	5	21.7%	0	0.0%	1	9.1%	0	0.0%	0	0.0%	0	0.0%
51–60 years	4	25.0%	4	33.3%	14	24.1%	1	50.0%	6	26.1%	2	33.3%	1	9.1%	0	0.0%	2	9.5%	2	11.8%
61–70 years	4	25.0%	3	25.0%	11	19.0%	0	0.0%	4	17.4%	1	16.7%	2	18.2%	0	0.0%	3	14.3%	7	41.2%
71–80 years	4	25.0%	2	16.7%	9	15.5%	1	50.0%	4	17.4%	1	16.7%	5	45.5%	1	50.0%	9	42.9%	5	29.4%
81–90 years	2	12.5%	2	16.7%	9	15.5%	0	0.0%	2	8.7%	1	16.7%	1	9.1%	1	50.0%	7	33.3%	2	11.8%
Occupation (n = 136)	Bricklayer	1	7.7%	0	0.0%	0	0.0%	0	0.0%	3	17.6%	0	0.0%	0	0.0%	0	0.0%	0	0.0%	0	0.0%
Miner	0	0.0%	1	11.1%	0	0.0%	0	0.0%	0	0.0%	0	0.0%	0	0.0%	0	0.0%	0	0.0%	0	0.0%
Retired	3	23.1%	4	44.4%	13	27.1%	1	50.0%	6	35.3%	1	25.0%	7	77.8%	2	100.0%	13	76.5%	5	33.3%
Housewife	4	30.8%	1	11.1%	16	33.3%	0	0.0%	2	11.8%	0	0.0%	0	0.0%	0	0.0%	3	17.6%	6	40.0%
Student	0	0.0%	0	0.0%	2	4.2%	0	0.0%	1	5.9%	0	0.0%	0	0.0%	0	0.0%	0	0.0%	1	6.7%
Doctor	0	0.0%	0	0.0%	2	4.2%	0	0.0%	0	0.0%	0	0.0%	0	0.0%	0	0.0%	0	0.0%	0	0.0%
Driver	0	0.0%	0	0.0%	1	2.1%	0	0.0%	1	5.9%	0	0.0%	0	0.0%	0	0.0%	0	0.0%	0	0.0%
Employee	1	7.7%	1	11.1%	5	10.4%	0	0.0%	1	5.9%	0	0.0%	0	0.0%	0	0.0%	0	0.0%	1	6.7%
Self-employed	3	23.1%	0	0.0%	5	10.4%	1	50.0%	2	11.8%	2	50.0%	0	0.0%	0	0.0%	1	5.9%	2	13.3%
Teacher	1	7.7%	2	22.2%	4	8.3%	0	0.0%	1	5.9%	1	25.0%	2	22.2%	0	0.0%	0	0.0%	0	0.0%
Birthplace (n = 146)	Coast	10	76.9%	3	33.3%	38	76.0%	2	100.0%	13	61.9%	5	83.3%	6	75.0%	1	50.0%	8	40.0%	11	73.3%
Highlands	3	23.1%	5	55.6%	10	20.0%	0	0.0%	7	33.3%	1	16.7%	2	25.0%	1	50.0%	10	50.0%	4	26.7%
Jungle	0	0.0%	1	11.1%	2	4.0%	0	0.0%	1	4.8%	0	0.0%	0	0.0%	0	0.0%	2	10.0%	0	0.0%
Tobacco (n = 59)	No	1	25.0%	3	75.0%	18	72.0%	2	100.0%	3	60.0%	1	50.0%	2	50.0%	1	100.0%	6	75.0%	3	75.0%
Yes	3	75.0%	1	25.0%	7	28.0%	0	0.0%	2	40.0%	1	50.0%	2	50.0%	0	0.0%	2	25.0%	1	25.0%
Alcohol (n = 57)	No	4	100.0%	3	100.0%	14	58.3%	2	100.0%	3	60.0%	1	50.0%	3	75.0%	1	100.0%	6	75.0%	3	75.0%
Yes	0	0.0%	0	0.0%	10	41.7%	0	0.0%	2	40.0%	1	50.0%	1	25.0%	0	0.0%	2	25.0%	1	25.0%
Hospital (n = 168)	Level III-1	7	43.8%	2	16.7%	12	20.7%	0	0.0%	4	17.4%	0	0.0%	1	9.1%	0	0.0%	2	9.5%	9	52.9%
Level III-2	9	56.3%	10	83.3%	46	79.3%	2	100.0%	19	82.6%	6	100.0%	10	90.9%	2	100.0%	19	90.5%	8	47.1%
Year of detection (n = 168)	2013	0	0.0%	0	0.0%	3	5.2%	0	0.0%	1	4.3%	0	0.0%	0	0.0%	1	50.0%	2	9.5%	0	0.0%
2014	1	6.3%	0	0.0%	3	5.2%	0	0.0%	0	0.0%	0	0.0%	0	0.0%	0	0.0%	0	0.0%	1	5.9%
2015	1	6.3%	1	8.3%	6	10.3%	0	0.0%	2	8.7%	0	0.0%	1	9.1%	0	0.0%	0	0.0%	3	17.6%
2016	2	12.5%	2	16.7%	5	8.6%	0	0.0%	1	4.3%	0	0.0%	1	9.1%	0	0.0%	2	9.5%	2	11.8%
2017	0	0.0%	1	8.3%	3	5.2%	1	50.0%	7	30.4%	0	0.0%	1	9.1%	0	0.0%	2	9.5%	1	5.9%
2018	4	25.0%	1	8.3%	7	12.1%	0	0.0%	1	4.3%	2	33.3%	1	9.1%	1	50.0%	6	28.6%	1	5.9%
2019	2	12.5%	2	16.7%	12	20.7%	1	50.0%	3	13.0%	1	16.7%	5	45.5%	0	0.0%	1	4.8%	1	5.9%
2020	3	18.8%	1	8.3%	7	12.1%	0	0.0%	2	8.7%	2	33.3%	0	0.0%	0	0.0%	1	4.8%	3	17.6%
2021	1	6.3%	3	25.0%	7	12.1%	0	0.0%	3	13.0%	0	0.0%	1	9.1%	0	0.0%	3	14.3%	1	5.9%
2022	2	12.5%	1	8.3%	5	8.6%	0	0.0%	3	13.0%	1	16.7%	1	9.10%	0	0.0%	4	19.0%	4	23.5%


OSCC was mostly characterized as well- or moderately differentiated (97.4%). A moderately differentiated grade was more frequent in individuals aged > 51 years (18.4 to 23.8%) vs younger persons (1.3 to 7.9%) and in retirees (42.9%) vs the employed (1.6 to 20.6%). A well-differentiated grade was more frequent in patients from 51 to 80 years of age (21.1 to 25%) vs other ages (5.3 to 11.8%) and in retirees (41.4%) vs the employed (1.7 ‒ 25.9%) (Table 3).

**Table 3:** Distribution of the degree of differentiation of oral squamous cell carcinoma according to the demographic and health characteristics of the study sample

Variables	Items	Poorly differentiated	Moderately differentiated	Well differentiated
n	%	n	%	n	%
Total (n = 156)		4	2.6%	76	48.7%	76	48.7%
Sex (n = 156)	Male	4	100.0%	32	42.1%	34	44.7%
Female	0	0.0%	44	57.9%	42	55.3%
Age (n = 156)	20-30 years	0	0.0%	1	1.3%	4	5.3%
31-40 years	0	0.0%	2	2.6%	4	5.3%
41-50 years	3	75.0%	6	7.9%	9	11.8%
51-60 years	1	25.0%	18	23.7%	16	21.1%
61-70 years	0	0.0%	18	23.7%	16	21.1%
71-80 years	0	0.0%	14	18.4%	19	25.0%
81-90 years	0	0.0%	17	22.4%	8	10.5%
Occupation (n = 125)	Bricklayer	2	50.0%	1	1.6%	1	1.7%
Miner	0	0.0%	1	1.6%	0	0.00%
Retired	0	0.0%	27	42.9%	24	41.4%
Housewife	0	0.0%	13	20.6%	15	25.9%
Student	0	0.0%	0	0.0%	3	5.2%
Doctor	0	0.0%	2	3.2%	0	0.0%
Driver	0	0.0%	2	3.2%	0	0.00%
Employee	1	25.0%	4	6.3%	4	6.9%
Self-employed	0	0.0%	8	12.7%	7	12.1%
Teacher	1	25.0%	5	7.9%	4	6.9%
Birthplace (n = 135)	Coast	3	75.0%	40	62.5%	48	71.6%
Highlands	1	25.0%	21	32.8%	16	23.9%
Jungle	0	0.0%	3	4.7%	3	4.5%
Tobacco (n = 55)	No	1	50.0%	22	84.6%	16	59.3%
Yes	1	50.0%	4	15.4%	11	40.7%
Alcohol (n = 54)	No	1	50.0%	20	80.0%	16	59.3%
Yes	1	50.0%	5	20.0%	11	40.7%
Hospital (n = 156)	Level III-1	0	0.0%	10	13.2%	17	22.4%
Level III-2	4	100.0%	66	86.8%	59	77.6%
Year of detection (n = 156)	2013	0	0.0%	2	2.6%	5	6.6%
2014	0	0.0%	2	2.6%	3	3.9%
2015	1	25.0%	7	9.2%	3	3.9%
2016	0	0.0%	6	7.9%	7	9.2%
2017	2	50.0%	6	7.9%	8	10.5%
2018	0	0.0%	12	15.8%	12	15.8%
2019	0	0.0%	10	13.2%	16	21.1%
2020	0	0.0%	11	14.5%	5	6.6%
2021	1	25.0%	9	11.8%	8	10.5%
2022	0	0.0%	11	14.5%	9	11.8%


Male patients had a higher probability of palatal OSCC (PR = 2.77; 95% CI: 1.20‒6.37) and a lower probability of well differentiated and moderately differentiated OSCC (PR = 0.943; 5% CI: 0.89–0.999) (p ≤ 0.39). Patients > 50 years of age had a higher probability of OSCC (PR = 1.54; 95% CI: 1.10‒2.14) located on the lip (PR = 5.28; 5% CI: 0.742–37.5) and well- or moderately differentiated OSCC (PR = 1.11; 5% CI: 0.977–1.25) (p ≤ 0.21). Retired persons had a lower probability of tongue-related OSCC (PR = 0.354; 95% CI: 0.174–0.72) and a higher probability of lip-related OSCC (PR = 5.52; 5% CI: 1.94 – 15.80) (p ≤ 0.005). Patients born in the highlands had a higher probability of lip-related OSCC (PR = 2.4; 95% CI: 1.12–5.10; p = 0.04) (Table 4).

**Table 4:** Probability of location and degree of differentiation of oral squamous cell carcinoma according to the demographic and health characteristics of the study sample

Variables	Presence	Tongue	Palate	Lip	Well differentiated	Well and Moderately differentiated
Yes	No	Yes	No	Yes	No	Yes	No	Yes	No	Yes	No
n (%)	n (%)	n (%)	n (%)	n (%)	n (%)	n (%)	n (%)	n (%)	n (%)	n (%)	n (%)
Sex	Male (Reference)	77 (40.7%)	112 (59.3%)	34 (44.7%)	42 (55.3%)	16 (21.1%)	60 (78.9%)	10 (13.2%)	66 (86.8%)	34 (48.6%)	36 (51.4%)	66 (94.3%)	4 (5.7%)
Female	92 (40.5%)	135 (59.5)	52 (56.5%)	40 (43.5%)	7 (7.6%)	85 (92.4%)	13 (14.1%)	79 (85.9%)	42 (48.8%)	44 (51.2%)	86 (100%)	0 (0%)
p-value	>0.999	0.163	0.014*	>0.999	>0.999	0.039*
PR [95% CI]	NA	NA	2.77 [1.20 – 6.37]	NA	NA	0.943 [0.89 – 0.999]
Age (years)	51-90 (Reference)	140 (44.6%)	174 (55.4%)	68 (48.9%)	71 (51.1%)	16 (11.5%)	123 (88.5%)	23 (16.5%)	116 (83.5%)	59 (46.5%)	17 (58.6%)	126 (99.2%)	1 (0.8%)
20-50	29 (29%)	71 (71%)	18 (62.1%)	11 (37.9%)	7 (24.1%)	22 (75.9%)	0 (0%)	29 (100%)	68 (53.5%)	12 (41.4%)	26 (89.7%)	3 (10.3%)
p-value	0.007*	0.225	0.081	0.015*	0.304	0.021*
PR [95% CI]	1.54 [1.10 – 2.14]	NA	NA	5.28 [0.742 – 37.50]†	NA	1.11 [0.977 – 1.25]
Retiree	Yes (Reference)	55 (45.1%)	67 (54.9%)	20 (36.4%)	35 (63.6%)	6 (10.9%)	49 (89.1%)	15 (27.3%)	40 (72.7%)	24 (47.1%)	27 (52.9%)	51 (100%)	0 (0%)
No	82 (42.7%)	110 (57.3%)	50 (61.7%)	31 (38.3%)	11 (13.6%)	70 (86.4%)	4 (4.9%)	77 (95.1%)	34 (45.9%)	40 (54.1%)	70 (94.6%)	4 (5.4%)
p-value	0.727	0.005*	0.793	<0.001*	>0.999	0.145
PR [95% CI]	NA	0.354 [0.174 – 0.72]	NA	5.52 [1.94 – 15.80]	NA	NA
House-wife	Yes (Reference)	32 (40%)	48 (60%)	21 (65.6%)	11 (34.4%)	2 (6.3%)	30 (93.8%)	3 (9.4%)	29 (90.6%)	15 (53.6%)	13 (46.4%)	28 (100%)	0 (0%)
No	105 (44.9%)	129 (55.1%)	49 (47.1%)	55 (52.9%)	15 (14.4%)	89 (85.6%)	16 (15.4%)	88 (84.6%)	43 (44.3%)	54 (55.7%)	93 (95.9%)	4 (4.1%)
p-value	0.514	0.073	0.359	0.562	0.4	0.574
PR [95% CI]	NA	NA	NA	NA	NA	NA
Birth-place	Highlands (Reference)	43 (43%)	57 (57%)	18 (41.9%)	25 (58.1%)	7 (16.3%)	36 (83.7%)	11 (25.6%)	32 (74.4%)	16 (42.1%)	22 (57.9%)	37 (97.4%)	1 (2.6%)
Coast – jungle	104 (39.5%)	159 (60.5%)	54 (52.4%)	49 (47.6%)	14 (13.6%)	89 (86.4%)	11 (10.7%)	92 (89.3%)	51 (52.6%)	46 (47.4%)	94 (96.6%)	3 (3.1%)
p-value	0.552	0.279	0.626	0.04*	0.339	>0.999
PR [95% CI]	NA	NA	NA	2.4 [1.12 – 5.10]	NA	NA
Tobacco	Yes (Reference)	20 (50%)	20 (50%)	11 (57.9%)	8 (42.1%)	2 (10.5%)	17 (89.5%)	2 (10.5%)	17 (89.5%)	11 (68.8%)	5 (31.3%)	15 (93.8%)	1 (6.3%)
No	40 (40.4%)	59 (59.6%)	22 (55%)	18 (45%)	3 (7.5%)	37 (92.5%)	7 (17.5%)	33 (82.5%)	16 (41%)	23 (59%)	38 (97.4%)	1 (2.6%9
p-value	0.346	>0.999	0.653	0.704	0.08	0.501
PR [95% CI]	NA	NA	NA	NA	NA	NA
Alcohol	Yes (Reference)	17 (44.7%)	21 (55.3%)	10 (58.8%)	7 (41.2%)	2 (11.8%)	15 (88.2%)	2 (11.8%)	15 (88.2%)	11 (64.7%)	6 (35.3%)	16 (94.1%)	1 (5.9%)
No	41 (42.7%)	55 (57.3%)	21 (52.5%)	19 (47.5%)	3 (7.5%)	37 (92.5%)	7 (17.5%)	33 (82.5%)	16 (43.2%)	21 (56.8%)	36 (97.3%)	1 (2.7%)
p-value	0.849	0.774	0.629	0.71	0.241	0.535
PR [95% CI]	NA	NA	NA	NA	NA	NA
Hospital	Level III-2 (Reference)	131 (43.2%)	172 (56.8%)	65 (49.6%)	66 (50.4%)	19 (14.5%)	112 (85.5%)	21 (16%)	110 (84%)	59 (45.7%)	70 (54.3%9	125 (96.9%)	4 (3.1%)
Level III-1	38 (33.6%)	75 (66.4%)	21 (56.8%)	16 (43.2%)	4 (10.8%)	33 (89.2%)	2 (5.4%)	35 (94.6%)	17 (63%)	10 (37%)	27 (100%)	0 (0%)
p-value	0.092	0.463	0.787	0.111	0.138	>0.999
PR [95% CI]	NA	NA	NA	NA	NA	NA
Year	2020-2022 (Reference)	59 (44.4%)	74 (55.6%)	30 (50.8%)	29 (49.2%)	8 (13.6%)	51 (86.4%9	8 (13.6%)	51 (86.4%)	22 (40.7%)	32 (59.3%)	53 (98.1%9	1 (1.9%)
2013-2019	110 (38.9%)	173 (61.1%)	56 (51.4%)	53 (48.6%)	15 (13.8%)	94 (86.2%)	15 (13.8%)	94 (86.2%)	54 (52.9%)	48 (47.1%)	99 (97.1%)	3 (2.9%)
p-value	0.335	>0.999	>0.999	>0.999	0.179	>0.999
PR [95% CI]	NA	NA	NA	NA	NA	NA
No unrecorded data were counted. Fisher’s exact test. PR: prevalence ratio. CI: confidence interval. †Correction for obtaining the PR by increasing one case in each cell.

## DISCUSSION 

Among the 416 medical records of patients treated at two specialized hospitals in the capital of Peru over the 10-year period from 2019 to 2022, 169 cases of OSCC were found, representing a prevalence of OSCC of 40.6%. This prevalence is comparable to that of other studies reporting ranges of 31.3% to 50.1% in Nigeria, Tanzania, Nepal, Pakistan, Saudi Arabia, the United Arab Emirates, and Romania.^5,14,20,32,36,41,43^ In contrast, this prevalence is higher than the range of 0.1% to 3.9% described in Mexico, Brazil, South Africa and Iraq,^1,6,7,30^ and lower than the 78% to 92.5% of OSCC reported in Canada, Morocco, India, Fiji Islands, and New Zealand.^16,21,23,24^ Nonetheless, it should be noted that comparisons with our study may be limited due to methodological differences, such as the provenance of the national level data,^1,4,6,18,21,22,24,50^ private/university/institute centers,^7,25,33,41,46^ and review periods.^5,7,13,25,23,33,36,37,40,46^

On the other hand, given that the majority of OC are OSCC, analysis of GLOBOCAN data from 2022 shows that the five-year prevalence of OC cases was distributed across continents as follows: Asia (62.48%), Europe (18.5%), the Americas (14.8%), Africa (3.1%), and Oceania (1.2%). However, this OC ranking changes when considering incidence based on age-standardized rates (ASR), revealing a group of 37 most affected countries (≥3.9 per 100,000), located primarily in Oceania, North America (United States and Canada), South and Southeast Asia, almost all of Europe, the Caribbean (Cuba and Saint Lucia), and Africa (Namibia and Botswana). Peru was included in the fourth group, along with Colombia (ASR: 1.7 to 2.5 per 100,000).^26^ These regional variations could be attributed to higher rates of smoking (Asia and Europe) and alcohol consumption (Europe and Oceania), as well as greater exposure to ultraviolet radiation (Oceania, Europe, and North America) and HPV infections (Africa, Asia, and the Caribbean).^2,22,26^

High rates of malignant transformation of the tongue have been reported, and it is the most common site of oral leukoplakia, which may explain its vulnerability to OSCC.^12,19^ We found that more than half of the cases of OSCC were of the tongue, which is also the most frequent localization reported by most previous studies, with comparable ranges of 41.2% to 73.4% in Oceania (Australia, Fiji Islands, and New Zealand),^21,44^ 34.3% to 64.2% in Asia (China, Indonesia, Japan, Nepal, Qatar, and the United Arab Emirates),^5,9,18,20,40,46^ 37.4% to 52.7% in the Americas (Argentina, Chile, Costa Rica, Mexico, Peru, Uruguay, United States, and Venezuela)^25,30,50^ and 23.2% to 51% in Europe (Finland, Netherlands, Romania, Spain, and Sweden).^4,13,14,19,33^ This information was confirmed in the CDC report for the period 2022 to 2024 in Lima, which showed that the percentage of OC of the tongue remained the highest, accounting for 50% to 62.5% of all OC.^15^ In contrast, in Africa, OSCC of the tongue was the most prevalent only in Tanzania and Uganda and was the second most frequent site of OSCC in Morocco, Nigeria, and Sudan, being described in a smaller proportion than in the present study (9.2% to 34.5%).^3,16,34,36,43^

In the present study, the lateral border of the tongue was affected in more than one-third of the cases of OSCC and affected almost 7 out of 10 cases of tongue OSCC. This location also proved to be the most frequently affected in Argentina, Chile, Costa Rica, Peru, and Uruguay, and the second most affected in Brazil and Venezuela with comparable ranges of 11.3% to 35.4%.^25^ On the other hand, we found that the dorsum of the tongue was the second most affected area in tongue OSCC, making up 20% of the cases. This contrasts with results from Latin America as described in a systematic review.^25^ Unfortunately, studies from other regions did not record OSCC by tongue subzones, thereby limiting comparisons. It is possible that the vulnerability of OSCC of the lateral and dorsal area of the tongue is associated with chronic trauma, infections from contact with teeth, bacteria or fungi, or lack of oral hygiene.^6,41,43^ The 2022 GLOBOCAN report lists Peru among the countries most affected by cancers attributable to HPV infections (ASR: 12.4 to 46.9).^26^

Certain areas of the oral cavity, such as the soft palate and oral mucosa, are lined with a thin non-keratinized epithelium allowing carcinogens to easily penetrate, thus yielding susceptibility to OSCC.^43^ The palate and oral mucosa were the next most prevalent locations of OSCC in the present study, although the ratio was only 1.4 or 1 in 10 cases, respectively. Analysis of multiple previous studies showed a range of palate OSCC involvement of 0.6% to 16.1%, being in the top three positions in sub-Saharan (Nigeria, Uganda, Tanzania),^34,36,43^ Arab (Morocco)^16^ and Latino (Venezuela, Costa Rica, Uruguay, Uruguay, Chile, Argentina) ethnicities.^25^ On the other hand, the range of oral mucosal OSCC involvement was 1.1% to 44.3% in India^23^ and Taiwan,^37^ the United Arab Emirates,^5^ Nepal,^20^ and Morocco,^16^ occupying the first position in the first two populations and the second place in the latter three.

The cells of highly differentiated OSCC resemble normal cells and thus are less specialized to facilitate disease extension.^43^ The Broder histologic grade was adopted by the World Health Organization and there is also a similar Bryne grading system. This criterion is corroborated with biopsy and is economically accessible but invasive.^14^ Almost all the OSCC of the present study were well-differentiated or moderately differentiated in equal proportions, similar to what has been reported in multiple previous studies, ranging from 80.2% to 95.4%, and being of similar grades,^9,13,18,25,32,41^ or with a predominance of well-differentiated^3,14,23,25,34,36,40,43^ and moderately differentiated OSCC.^30^ On the other hand, poorly differentiated OSCC was infrequent (2.6%) and undifferentiation was negligible, as has been found in several studies that included Peru, with ranges of ≤ 8.4%.^3,9,14,18,25,30,32,33,34,36,40,41,43,44,50^ Despite the low severity described in previous studies on OSCC, the degree of differentiation has minimal prognostic value compared to molecular testing.^43,47^

Women may show a greater interest in seeking oral healthcare compared to men.^25^ More than half of the cases of OSCC in this study affected females, as noted in a few previous studies including one from Peru, describing ranges of 54.7% to 69.6%.^21,23,25,32^ This was also confirmed by the CDC report for the period 2022 to 2024 in Lima, which stated that the percentage of women with OC had increased, accounting for 53.1% to 60% of all OC.^15^ A history of OSCC was found to be more frequent in men: 53.1% to 60%.^1,3-7,9,13,14,16,18-21,24,25,30,33,34, 36,37,40,41,43,44,46^ However, in the present study, gender had a no or weak association with the presence or differentiation of OSCC. Nonetheless, upon analysis of the location of cancer, the probability of males presenting OSCC in the tongue was almost double compared to females. This result could be due to men having a higher risk of tobacco and alcohol exposure or that men give less priority to oral health cthan do women.^20,22,41^

Older age is related to risk factors for OSCC, due to impaired defense mechanisms and a decreased ability to regenerate DNA damage with age.^6,19^ This study found a higher prevalence of OSCC in individuals > 50 years of age, with a ratio of 8.3:1 cases in agreement with most previous studies, which reported ranges of 54% to 92.7%^4,6,9,14,20,23,25,32,34,36,40,43,46,50^ and mean ages of approximately 50 to 66 years.^3,5,6,9,13,14,16,19- 21,23,25,30,33,34,36,37,40,43,44,46^ Younger patients tend to have a better survival rate for OSCC, but treatments may need to be extended due to more aggressive tumors characterized by low differentiation.^22^ In addition, we found age to be associated with the presence and differentiation of OSCC, with individuals over 50 years old having a 54% higher probability of presenting OSCC, as well as a 4.28 times higher likelihood of lip OSCC and 11% higher presentation of well-differentiated or moderately differentiated OSCC. According to the report by National Institute of Statistics and Informatics (INEI) for 2025 in Peru, 58.2% of the elderly population have comorbidities associated with metabolic disorders such as obesity, diabetes, and hypertension. Although the evidence is limited, high levels of body fat, blood sugar, and blood pressure have been described as possible risk factors for OC.^17,28^

Individuals who are retired are usually of older age, with the subsequent risk for the development of OSCC, and thus may seek healthcare more often.^20^ The majority of cases of OSCC in the present study were in retirees (4:1) and housewives (2.3:1). In contrast, only one study conducted in Brazil reported the prevalence of OSCC in active workers (7.6:1). The present study also found that employment was associated with the location of OSCC, with retirees being almost half as likely to have tongue OSCC and almost five times as likely to have lip OSCC compared to non-retired persons. The occupational data for the retired individuals in this study was not recorded in their medical records, which is a limitation of the study. However, 2025 INEI report in Peru indicated that 40% of retired older adults had been employed in agriculture, fishing, mining, and construction. These outdoor occupations could increase the risk of exposure to carcinogens such as solar ultraviolet radiation.^42^ On the other hand, OSCC in housewives primarily affected the lateral border of the tongue (50%) and occurred in women aged 60 to 80 years (56%). As previously explained, the lateral border of the tongue is a high-risk area for OC.^6,41,43^ According to CDC data for the period 2012 to 2024 in Peru, chronic diseases affected women (≈80%) more than men (≈70%) among adults aged 60 and older, which could be a risk factor for OC.^41^ An unhealthy diet high in fatty acids, sugars, and salt is associated with chronic systemic inflammation and can activate oncogenes, which may lead to the development of OC.^17,22^

Persons with OSCC require specialized care, leading to referral from provincial hospitals to hospitals with more specialized levels of care located in the capital (Lima). Most of the OSCC cases in this study were diagnosed in people born on the coast (6.7:1) and the highlands (2.9:1). It is likely that most of the study participants completed their primary education in their place of birth until the age of 16 and then migrated to larger cities, such as the capital of Peru. For decades, farming, ranching and fishing have remained common occupations in rural areas, particularly in the coastal provinces and highland regions of Peru, according to data from INEI for 2025 in Peru. These outdoor occupations could increase the risk of exposure to carcinogens such as solar ultraviolet radiation.^42^ Likewise, the probability of presenting with lip OSCC was found to be 1.5 times greater in subjects born in the highlands compared to patients born on the coast or the jungle. Furthermore, among all oral lesions, lip lesions are associated with the HPV, which could pose a potential risk of OC.^2,17,22,27^

In regard to other variables analyzed in this study, the prevalence of OSCC was shown to be altered by the COVID-19 pandemic, with a progressive increase being observed from 2013 to 2019 and a decrease from 2020 to 2022, probably due to the restrictions in healthcare during this period. Nonetheless, the prevalence of OSCC was not found to be associated with the restrictions related to healthcare during the pandemic. This can be corroborated by the CDC report on MINSA hospitals, which described a progressive increase in OC cases: 143 cases in 2021 (12.6% in Lima), 175 cases in 2022 (18.3% in Lima), 185 cases in 2023 (21.6% in Lima), and 220 cases in 2024 (19.1% in Lima).^15^

Regarding lifestyle factors, neither alcohol nor tobacco were found to be associated with OSCC, in concordance with several previous studies, including one from Peru which reported infrequent ranges of 54.7% to 98.8%,^3,9,13,16,19,25,32,41^ but contrasted with others that reported ranges of 63.5% to 90%,^4,7,14,23,34,37^ indicating that these toxic habits may be common risk factors for OSCC. According to the GLOBOCAN report, Peru was among the countries moderately affected by OC attributable to alcohol consumption (population attributable fraction: 18.3%).^26^ Multiple explanations for chronic inflammation and oral microbiome dysbiosis point to smoking as a risk factor for cancer, therefore, the results of this study should be interpreted with caution, as data on tobacco and alcohol consumption were missing in several records.^11,17,22,27^ On the topic of viral metagenomes, this study did not collect data, because viral infections were not frequently detected in patients with OC. However, the literature suggests that individuals infected with oncogenic viruses, such as Epstein-Barr, cytomegalovirus and HPV, may be at increased risk for OSCC progression, so that prophylactic vaccination is an appropriate preventive measure.^2,17^

According to the CDC for 2025 in Peru, approximately 80% of cancer cases in Peru are initially diagnosed based on clinical presentation, with the histology of the primary tumor serving as the basis for diagnosis. Histological analysis using a tumor biopsy is the traditional method for confirming the diagnosis of OC. However, its invasive nature is a point of debate, compared to other methods such as liquid biopsies, which offer screening options at early stages.^17,22,35^ Blood biopsy results showing high levels of systemic markers for neutrophils, lymphocytes, platelets, and monocytes indicate an inflammatory response due to periodontitis, and these markers could also be used as early indicators for OC.^48^ Saliva also appears to be a viable alternative to blood samples, with RNA-sequencing-based omics analysis yielding reliable results in the diagnosis of OSCC.^11^ Although based on limited evidence from observational studies, periodontitis seems to be associated with a higher risk of developing OSCC.^17,31^ Therefore, it is necessary to treat chronic inflammation and prevent the transition to acute inflammation in order to avoid the development of a pro-tumorigenic microenvironment for OC.^45,51^

According to data from the GLOBOCAN in 2022, 697 new cases of oral cavity and lip cancer were diagnosed in Peru (ASR: 6.1 per 100,000), resulting in approximately 294 deaths. Most OSCC lesions are detected at a late stage.^22,26^ Early detection of OSCC may be associated with greater access to dental care and greater diagnostic competence of the health personnel. Countries with medium-low status of development, such as Peru, may have limited health education. This could result in late detection of OSCC with survival rates of 23% ‒ 57% compared to the 60% found in developed countries.^11,14,20^ The results of this study show the relevant epidemiological characteristics that should be taken into account in diagnostic decision making and prognostic factors for providing the most adequate treatment management. It is important for dentists to be properly trained to identify suspicious lesions for referral to specialized centers.^22^

This study has some limitations. The Peruvian national healthcare system is currently being digitalized, leading to selection bias due to problems in achieving access to very old medical records or those of deceased persons, making data collection difficult. Multiple records were found to be incomplete in relation to relevant data on risk factors, which should also be included in digital medical records. Longitudinal studies are necessary to validate demographic and lifestyle risk factors, as well as their association with other biological factors such as oral microbiota and oncogenic viruses.^11,17^

## CONCLUSION 

Taking into account the limitations of this study, the following conclusions were reached:

The incidence of OSCC has progressively increased since 2013 at two public hospitals in Peru.
OSCC was found to be more frequent on the tongue and were well- or moderately differentiated.
OSCC is more common in individuals over 50 years of age, retirees, and those born in coastal areas.
Age over 50 was associated with a higher likelihood of OSCC, particularly when located on the lip and with a good or moderate degree of differentiation.
Male sex was associated with a higher likelihood of OSCC of the palate.
Retirees and those born in the highlands were associated with a higher likelihood of OSCC on the lip.

These findings highlight the important role of dentists in identifying the presence and location of OSCC during clinical evaluation, and contribute to clarifying its association with demographic factors such as sex, age, employment status, and region of birth.

## ACKNOWLEDGMENTS

The authors express their gratitude to the Statistics Departments for the support in obtaining study data in the Santa Rosa Hospital and Guillermo Almenara Irigoyen Hospital in Peru.

## Appendix

**Appendix Table A1** Results of articles regarding oral squamous cell carcinoma (https://doi.org/10.6084/m9.figshare.28924784)

## References

[ref1] Abram MH, van Heerden WF, Rheeder P, Girdler-Brown BV, van Zyl AW (2012). Epidemiology of oral squamous cell carcinoma. SADJ.

[ref2] Aghaeipour F, Salehiniya H, Abbaszadeh H (2021). Prevalence of human papillomavirus (HPV) in oral mucosal lesions in Iran: A systematic review and meta-analysis. J Med Virol.

[ref3] Alim N, Elsheikh M, Satti AA, Tabassum N, Suleiman AM (2024). Recurrence of oral squamous cell carcinoma in surgically treated patients at Khartoum Teaching Dental Hospital retrospective cross-sectional study. BMC Cancer.

[ref4] Al-Jamaei AAH, van Dijk BAC, Helder MN, Forouzanfar T, Leemans CR, de Visscher JGAM (2022). A population-based study of the epidemiology of oral squamous cell carcinoma in the Netherlands 1989–2018, with emphasis on young adults. Int J Oral Maxillofac Surg.

[ref5] Al-Rawi NH, Hachim IY, Hachim MY, Salmeh A, Uthman AT, Marei H (2023). Anatomical landscape of oral squamous cell carcinoma: A single cancer center study in UAE. Heliyon.

[ref6] Alshami ML, Al-Maliky MA, Alsagban AA, Alshaeli AJ (2023). Epidemiology and incidence of oral squamous cell carcinoma in the Iraqi population over 5 years (2014-2018). Health Sci Rep.

[ref7] Alves AM, Correa MB, Silva KDD, Araújo LMA, Vasconcelos ACU, Gomes APN (2017). Demographic and clinical profile of oral squamous cell carcinoma from a service-based population. Braz Dent J.

[ref8] Astekar M, Taufiq S, Sapra G, Agarwal A, Murari A, Putthia H (2018). Prevalence of oral squamous cell carcinoma in Bareilly Region: A seven year institutional study. J Exp Ther Oncol.

[ref9] Bai XX, Zhang J, Wei L (2020). Analysis of primary oral and oropharyngeal squamous cell carcinoma in inhabitants of Beijing, China-a 10-year continuous single-center study. BMC Oral Health.

[ref10] Bald X (2014). Oral complications of head and neck radiotherapy and chemotherapy treatments. Rev Cient Odontol.

[ref11] Belibasakis GN, Seneviratne CJ, Jayasinghe RD, Vo PT, Bostanci N, Choi Y (2024). Bacteriome and mycobiome dysbiosis in oral mucosal dysplasia and oral cancer. Periodontol 2000.

[ref13] Capote-Moreno A, Brabyn P, Muñoz-Guerra MF, Sastre-Pérez J, Escorial-Hernandez V, Rodríguez-Campo FJ (2020). Oral squamous cell carcinoma: epidemiological study and risk factor assessment based on a 39-year series. Int J Oral Maxillofac Surg.

[ref14] Carp A, Nicolau A, Moscalu M, Popescu E (2022). Predictive factors in the appearance and evolution of squamous cell carcinomas of the oral cavity. Medicine.

[ref18] Elaiwy O, El Ansari W, AlKhalil M, Ammar A (2020). Epidemiology and pathology of oral squamous cell carcinoma in a multi-ethnic population: Retrospective study of 154 cases over 7 years in Qatar. Ann Med Surg.

[ref20] Gajurel R, Gautam DK, Pun CB, Dhakal HP, Petrovski BÉ, Costea DE (2020). Trends and clinicopathological characteristics of oral squamous cell carcinomas reported at a tertiary cancer hospital in Nepal during 1999 to 2009. Clin Exp Dent Res.

[ref21] Gavidi R, Rich A, Cox B, King T (2014). Comparing the occurrence of oral squamous cell carcinoma in New Zealand and the Fiji Islands from 2000-2010. Int J Cancer Res.

[ref22] Ghanem AS, Memon HA, Nagy AC (2024). Evolving trends in oral cancer burden in Europe: a systematic review. Front Oncol.

[ref23] Ghatage DD, Kendre AG, Palve DH, Dhobley A, Ghodichor D, Lakawath A (2024). Oral squamous cell carcinoma: A 26-year institutional cross-sectional study. J Oral Maxillofac Pathol.

[ref24] Ghazawi FM, Lu J, Savin E, Zubarev A, Chauvin P, Sasseville D (2020). Epidemiology and patient distribution of oral cavity and oropharyngeal SCC in Canada. J Cutan Med Surg.

[ref25] Gilligan G, Panico R, Lazos J, Morelatto R, Belardinelli P, Criscuolo MI (2024). Oral squamous cell carcinomas and oral potentially malignant disorders: A Latin American study. Oral Dis.

[ref27] Golusiński W, Golusińska-Kardach E, Machczyński P, Szewczyk M (2025). HPV-driven head and neck cancer: the european perspective. Viruses.

[ref28] Gormley M, Dudding T, Thomas SJ, Tyrrell J, Ness AR, Pring M (2023). Evaluating the effect of metabolic traits on oral and oropharyngeal cancer risk using Mendelian randomization. Elife.

[ref29] Graillon N, Iocca O, Carey RM, Benjamin K, Cannady SB, Hartner L (2022). What has the National Cancer Database taught us about oral cavity squamous cell carcinoma. Int J Oral Maxillofac Surg.

[ref32] Idris A, Vani N, Saleh S, Tubaigy F, Alharbi F, Sharwani A (2016). Relative frequency of oral malignancies and oral precancer in the biopsy service of Jazan Province, 2009-2014. Asian Pac J Cancer Prev.

[ref33] Jäwert F, Nyman J, Olsson E, Adok C, Helmersson M, Öhman J (2021). Regular clinical follow-up of oral potentially malignant disorders results in improved survival for patients who develop oral cancer. Oral Oncol.

[ref34] Kakande E, Byaruhaga R, Kamulegeya A (2010). Head and neck squamous cell carcinoma in a Ugandan population: A descriptive epidemiological study. J Afr Cancer.

[ref35] Kinane DF, Gabert J, Xynopoulos G, Guzeldemir-Akcakanat E (2024). Strategic approaches in oral squamous cell carcinoma diagnostics using liquid biopsy. Periodontol 2000.

[ref37] Lee YC, Young CK, Chien HT, Chin SC, Iandelli A, Liao CT (2021). Characteristics and outcome differences in male and female oral cavity cancer patients in Taiwan. Medicine.

[ref38] Markopoulos AK (2012). Current aspects on oral squamous cell carcinoma. Open Dent J.

[ref39] Melo-Alvim C, Neves ME, Santos JL, Abrunhosa-Branquinho AN, Barroso T, Costa L (2022). Radiotherapy, chemotherapy and immunotherapy-current practice and future perspectives for recurrent/metastatic oral cavity squamous cell carcinoma. Diagnostics.

[ref40] Rahadiani N, Habiburrahman M, Stephanie M, Handjari DR, Krisnuhoni E (2023). Estimated projection of oral squamous cell carcinoma annual incidence from twenty years registry data: a retrospective cross-sectional study in Indonesia. PeerJ.

[ref41] Saira; Ahmed R, Malik S, Khan MF, Khattak MR (2019). Epidemiological and clinical correlates of oral squamous cell carcinoma in patients from north-west Pakistan. J Pak Med Assoc.

[ref42] Slavinsky V, Helmy J, Vroman J, Valdebran M (2024). Solar ultraviolet radiation exposure in workers with outdoor occupations: a systematic review and call to action. Int J Dermatol.

[ref43] Sohal KS, Owibingire SS, Moshy JR, Deoglas DK, Laizer PJ, Kalyanyama BM (2022). Orofacial squamous cell carcinoma: Analysis of histopathological reports of 465 patients in Tanzania. Clin Cancer Investig J.

[ref44] Sun A, Sharma D, Choi SW, Ramamurthy P, Thomson P (2023). Oral cancer in Australia: Rising incidence and worsening mortality. J Oral Pathol Med.

[ref46] Tran CM, Kuroshima T, Oikawa Y, Michi Y, Kayamori K, Harada H (2021). Clinicopathological and immunohistochemical characteristics of pigmented oral squamous cell carcinoma. Oncol Lett.

[ref47] Vijayakumar G, Sharma G, Narwal A, Kamboj M (2021). Broder versus Bryne’s histologic grading parameters on incision biopsy specimens: A comparative study with P53 and KI67 expression. J Oral Maxillofac Pathol.

[ref48] Walther KA, Gröger S, Vogler JAH, Wöstmann B, Meyle J (2024). Inflammation indices in association with periodontitis and cancer. Periodontol 2000.

[ref49] Warnakulasuriya S, Kerr AR (2021). Oral cancer screening: past, present, and future. J Dent Res.

[ref50] Yang J, Guo K, Zhang A, Zhu Y, Li W, Yu J (2023). Survival analysis of age-related oral squamous cell carcinoma: a population study based on SEER. Eur J Med Res.

[ref51] Zhou Y, Meyle J, Groeger S (2024). Periodontal pathogens and cancer development. Periodontol 2000.

